# Exosomes from human umbilical cord mesenchymal stem cells promote the growth of human hair dermal papilla cells

**DOI:** 10.1371/journal.pone.0320154

**Published:** 2025-04-30

**Authors:** Yu-Cheng Chen, Wei-Cheng Tsai, Zhi-Xiang Li, Wan-Jung Lin, Hao-Yu Lin, Yi-Ju Hsieh, Kai-Hsuan Wang, You-Yan Chen, Tsong-Long Hwang, Tzou-Yien Lin

**Affiliations:** 1 Center for Drug Research and Development, College of Human Ecology, Chang Gung University of Science and Technology, Taoyuan, Taiwan; 2 Graduate Institute of Health Industry Technology, College of Human Ecology, Chang Gung University of Science and Technology, Taoyuan, Taiwan; 3 ExoOne Bio Co., Ltd., Taipei City, Taiwan; 4 School of Traditional Chinese Medicine, College of Medicine, Chang Gung University, Taoyuan, Taiwan; 5 Department of Chemical Engineering, Ming Chi University of Technology, New Taipei City, Taiwan; 6 Graduate Institute of Natural Products, College of Medicine, Chang Gung University, Taoyuan, Taiwan; 7 Department of Anesthesiology, Chang Gung Memorial Hospital, Taoyuan, Taiwan; 8 Department of Paediatrics, Chang Gung Memorial Hospital, Chang Gung University College of Medicine, Taoyuan, Taiwan; Northwestern University, UNITED STATES OF AMERICA

## Abstract

Human hair dermal papilla cells (HHDPCs) play a significant role in hair growth. This study found that human umbilical cord mesenchymal stem cell-derived exosomes (UC-MSC-Es) effectively enhanced cell growth of HHDPCs. UC-MSC-Es has a size range of 30–180 nm and expression of CD9, CD63, CD81, CD73, and TSG101. UC-MSC-Es significantly increased cell populations of HHDPCs in the S and G2/M phases. UC-MSC-Es also increased the expression of cell cycle-related proteins, β-catenin, and cyclin D1. Further mechanistic studies demonstrated that UC-MSC-Es promoted the phosphorylation of Akt and GSK-3β, and the inhibition of PI3K and Akt reduced the proliferative effects of UC-MSC-Es. Collectively, these findings suggest that UC-MSC-Es have a potential effect in treating hair loss through modulating PI3K and Akt-dependent pathways in HHDPCs.

## Introduction

A hair follicle is a tube-like structure in the skin that surrounds the root of a hair. The dermal papilla cells are essential for the hair growth cycle, which includes the anagen, catagen, and telogen phases [1,2]. The factors of hair loss are complex and include genetic factors [3], as well as mineral deficiencies [4], hormonal imbalances [5], and psychological stress [6]. The FDA has approved several therapeutic drugs for hair loss, including finasteride, minoxidil, and baricitinib [7,8]. Finasteride inhibits the conversion of testosterone to dihydrotestosterone [9], minoxidil enhances microcirculatory support to follicles [10], and baricitinib targets inflammatory pathways to promote hair regrowth [11]. The potential adverse side effects of these medications include decreased libido, erectile dysfunction, skin irritation, upper respiratory tract infections, and high blood pressure. Therefore, there is an urgent need for safer alternative treatments for hair loss.

Extracellular vesicles are categorized into four main classes based on their biogenesis, size, and protein markers: exosomes, microvesicles, oncosomes, and apoptotic bodies [12]. Exosomes are the smallest category, ranging from 30 to 200 nm. They play pivotal roles in regulating biological functions by transporting biomolecules, such as lipids, proteins, and nucleic acids [13]. In a clinical environment, exosomes can be utilized as disease biomarkers, therapeutic agents, and components of drug delivery systems, as well as in vaccine formulations [14]. Researchers dealing with hair restoration have investigated the therapeutic effects of exosomes derived from adipose-derived stem cells [15], dermal papilla cells, keratinocytes, human amniotic fluid stem cells, and myeloid-derived suppressor cells [16]. Exosomes derived from umbilical cord mesenchymal stem cells (UC-MSCs) have proven highly valuable in skin repair [17].

The PI3K/Akt signaling pathway is critical for maintaining the hair-inductive properties of human hair follicle dermal papilla cells (DPCs). Activation of this pathway enhances trichogenic gene expression and supports hair follicle and DPC growth [18]. Natural extracts from Butterfly Pea, Emblica Fruits, Kaffir Lime, Soybean, and Thunbergia Laurifolia have been shown to induce autophagy and enhance stemness markers, essential for DPC growth, via the Akt signaling pathway [19]. Additionally, micro-current electrical stimulation enhances hair follicle regeneration by activating the PI3K/Akt pathway [20]. The results highlight the crucial importance of the PI3K/Akt signaling pathway in regulating dermal papilla cell function and promoting hair follicle growth.

The current study examined the effects of UC-MSC-derived exosomes (UC-MSC-Es) on human hair dermal papilla cells (HHDPCs). UC-MSC-Es enhanced the proliferation of HHDPCs and modulated the phosphorylation of Akt and GSK-3β. PI3K and Akt inhibitors reversed the UC-MSC-Es-induced cell growth, suggesting that the proliferative effect may involve the PI3K and Akt signaling pathways. These findings provide important insights applicable to the development of treatments for hair loss.

## Materials and methods

### Materials

UC-MSC-Es were provided by Taiwan Bio Therapeutics Co (HSZ, TWN). Human hair dermal papilla cells (HHDPCs, Cat. 2400) were purchased from the Bioresource Collection and Research Center (TPE, TWN). Mesenchymal stem cell medium (MSCM, Cat. 7501), mesenchymal stem cell growth supplement (MSCGS, Cat. 7552), and penicillin/streptomycin solution (Cat. 0503) were obtained from ScienCell Research Laboratories (CA, USA). PI/RNase Staining Buffer (Cat. 550825) was obtained from BD Bioscience (NJ, USA). Pierce™ RIPA Buffer (Cat. 89901) and fetal bovine serum (FBS, Cat. 0025) were acquired from Thermo Fisher (MA, USA). Idelalisib (Cat. HY-13026) and MK-2206 dihydrochloride (Cat. HY-10358) were procured from MedChemExpress (NJ, USA). WST-1 (Cat. 11644807001) was purchased from Roche (Basel, Switzerland). Dimethyl sulfoxide (DMSO), phenylmethylsulfonyl fluoride (PMSF), and phosphatase inhibitors were purchased from Sigma-Aldrich (MO, USA). Protein assay dye reagent concentrate was obtained from BIORAD (CA, USA). Antibodies against Akt (Cat. 4691S), phospho-Akt (Ser473, Cat. 4060S), phospho-Akt (Thr308, Cat. 2965S), phospho-GSK3β (Cat. 9323), GSK3β (Cat. 12456), CD9 (Cat. 13174), TSG101 (Cat. 72312), and Calnexin (Cat. 2679) as well as horseradish peroxidase (HRP)-conjugated secondary antibodies were purchased from Cell Signaling (MA, USA). Antibodies against cyclin D1 (Cat. ab16663), CD63 (Cat. ab134045), (Cat. ab8226), and β-catenin (Cat. ab32572) were purchased from Abcam (Cambs, UK). Antibodies against CD81 (Cat. ExoAB-CD81A-1) were purchased from SBI (CA, USA). Antibodies against CD73 (Cat. 12231-1-AP) and GAPDH (Cat. 10494-1-AP) were purchased from Proteintech (IL, USA).

### Exosome isolation

UC-MSC cells (Taiwan Bio Therapeutics Co. Ltd., Hsinchu, Taiwan) were expanded in culture using exosome-depletion MEMα medium until reaching confluency of approximately 80%. The conditioned medium was then collected and filtered through 0.22 μM filters. Exosomes were subsequently isolated via Tangential Flow Filtration using a 300 kDa cassette (Sartorius, Goettingen, Germany).

### Nanoparticle tracking analysis

Nanoparticle tracking analysis is used to determine the size distribution of particles in fluids by analyzing the rate of Brownian motion using dynamic light scattering. Following TFF purification, the concentrated exosome solution was characterized using NanoSight NS300 (Malvern Panalytical Ltd., Malvern, UK). Briefly, samples were diluted in PBS to a final volume of 1 mL, with the concentration adjusted by observing a particle/frame rate of 30–80. Diluted samples were injected into the nanoparticle tracking analysis laser chamber to determine the particle size distribution in the concentrated exosome solution.

### Transmission electron microscopy analysis

Samples were fixed in 2.5% glutaraldehyde at 4 °C overnight to preserve their structural integrity. After fixation, the samples were thoroughly washed with PBS to eliminate any residual fixative. The samples were then placed onto formvar-coated carbon grids and allowed to adhere properly. Next, the grids were negatively stained with a 1% aqueous solution of phosphotungstic acid (pH 7.0) for 60 seconds to enhance contrast. Visualization was carried out using a transmission electron microscope (TEM) (Hitachi, Japan) operating at 80 kV to examine the morphology and size of the vesicles.

### Cell proliferation assay

HHDPCs were seeded in a 24-well plate at a density of 2 x 10^3^ cells per well and incubated at 37°C for 24 h. The cells were treated with either PBS, exosomes, or minoxidil and incubated in a 37°C incubator for 72 h. Following this incubation period, 20 μL of WST-1 was added to each well to assess cell proliferation and viability. This assay relies on converting the tetrazolium salt WST-1 into a colored formazan dye by mitochondrial dehydrogenase activity in viable cells. The cells were then incubated at 37°C for an additional 2 h. Absorbance readings were obtained using a spectrophotometer at 450 nm and 620 nm.

### Cell cycle analysis

HHDPCs were seeded onto a 6-well plate at a density of 2 x 10^4^ cells per well. Following incubation at 37°C for 24 h, The cells were treated with either PBS or exosomes in a 37°C incubator for 48 h. Following incubation, the cells were collected and centrifuged at 200 g for 8 min to remove the supernatant. After 70% ethanol was added, the samples were stored at -20 °C overnight. A second round of centrifugation was then performed to eliminate the supernatant. The resulting pellet was rinsed once using PBS before being stained with PI/RNase staining buffer for 10 min. Analysis was conducted using an Attune NxT Flow Cytometer (Waltham, MA, USA).

### Immunoblotting analysis

HHDPCs were seeded onto a 6-well plate at a density of 1 x 10^6^ cells per well after incubating at 37°C for 24 h. The cells were treated with either PBS or exosomes in a 37°C incubator for the specified duration. After incubation, the cells were collected, and the supernatant was discarded. The cells were then lysed with RIPA buffer containing 1 mM PMSF, 1% protease inhibitor cocktail, and 1% phosphatase inhibitor cocktail. The lysates were centrifuged at 19,000 g after sonication, and the supernatant was collected. The protein concentration was determined using a protein assay dye reagent. Western blotting was performed with the proteins equally distributed on a sodium dodecyl sulfate-polyacrylamide post-electrophoresis (SDS-PAGE) gel. Following electrophoresis, the proteins were transferred to nitrocellulose membranes. To minimize nonspecific binding, the membranes were incubated with a 5% skim milk solution in 0.1% TBST for 60 min, followed by TBST washes. The membranes were then exposed to specific primary antibodies (cyclin D1, β-catenin, Akt, phospho-Akt (Ser473), phospho-Akt (Thr308), phospho-GSK3β, GSK3β, and GAPDH) at 4°C overnight. After a second round of TBST washing, the cells were subjected to secondary antibodies attached to HRP-conjugated secondary antibodies for 60 min. The protein bands were visualized via the ChemiDoc MP Imaging System from BIORAD (CA, USA).

The identification of exosomes was performed via SDS-PAGE with equal quantities of proteins in each concentrated exosome sample. The concentrated exosome samples were then subjected to SDS-PAGE and transferred to PVDF membranes, which were then blocked in 5% BSA at room temperature for 1 h. After incubation with primary antibodies (CD9, CD63, CD81, CD73, TSG101, and Calnexin) at 4°C overnight, the membranes were washed and incubated with HRP-conjugated secondary antibodies at room temperature for 1 h. The protein bands were detected using a chemiluminescence system (Amersham Biosciences located in Piscataway Corp., NJ, USA) to identify UC-MSC-Es. The brightness of the bands was measured using a UVP Biospectrum imaging system (UVP, CA, USA).

### Statistical analysis

Statistical analyses were conducted with data expressed as the mean ± SEM. Student’s *t*-test was used to compare data from each group, analyzed using SigmaPlot (Systat Software, San Jose, CA, USA). Statistical significance was determined at a *p*-value of less than 0.05.

## Results

### Identification of UC-MSC-Es

**Fig 1:**
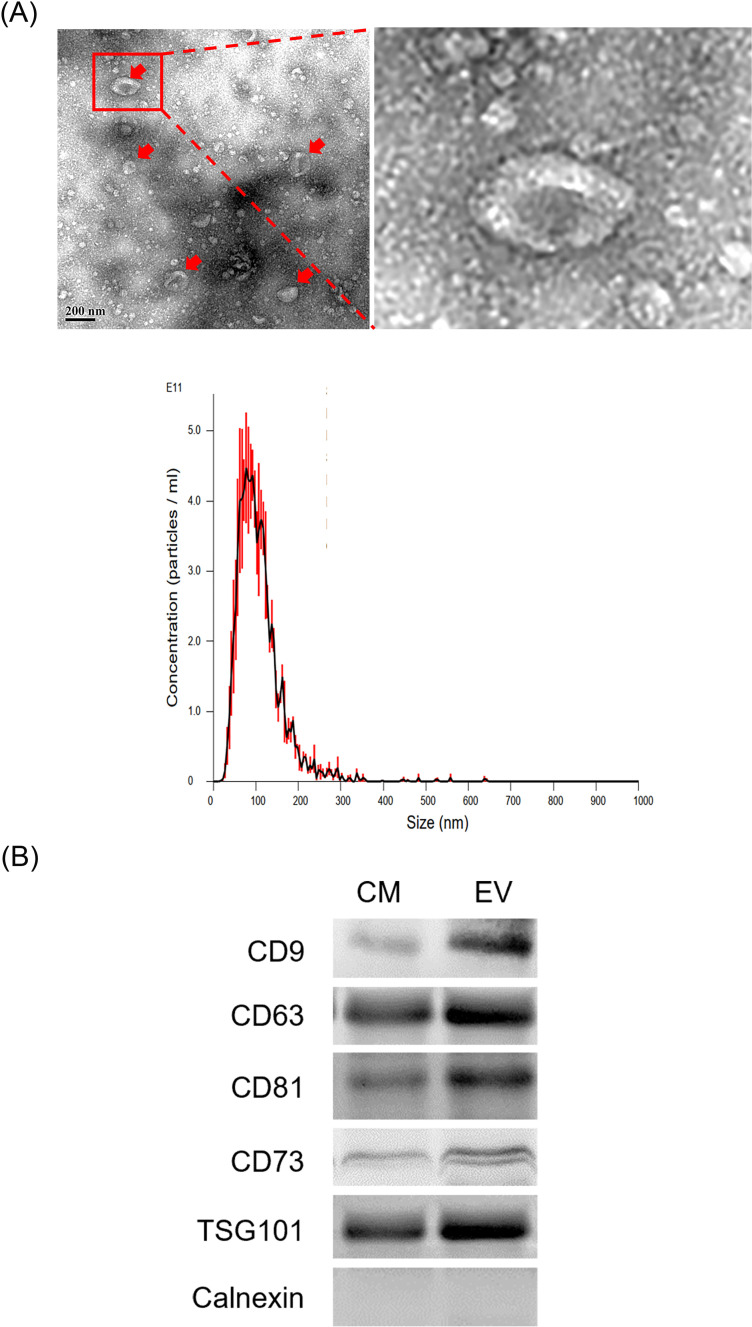
Identification of exosomes derived from UC-MSCs (UC-MSC-Es). (A) Exosome morphology and sizes were measured using transmission electron microscopy (up) and nanoparticle tracking analysis assay (down), respectively. (B) Protein expression levels of conditioned medium (CM) and extracellular vesicles (EV) were determined by western blotting. The results were conducted with three independent experiments (n = 4).

UC-MSC-Es were characterized using TEM, nanoparticle tracking analysis, and western blot analysis. TEM revealed intact, cup-shaped membrane vesicles, with particles ranging in size from 30 to 180 nm (Fig 1A). Western blot analysis revealed the expression of exosome-specific CD9, CD63, CD81, CD73, and TSG101 in concentrated, purified exosomes. Calnexin is a protein that resides in the endoplasmic reticulum and is typically not found in exosomes. In this study, calnexin was not detected in the conditioned medium and extracellular vesicles (Fig 1B). These results confirm that the extracellular vesicles are intact, purified exosomes that have been secreted into the extracellular environment. They are further characterized by their distinct size, morphology, and expression of specific markers.

### Influence of UC-MSC-Es on cellular proliferation in HHDPCs

**Fig 2:**
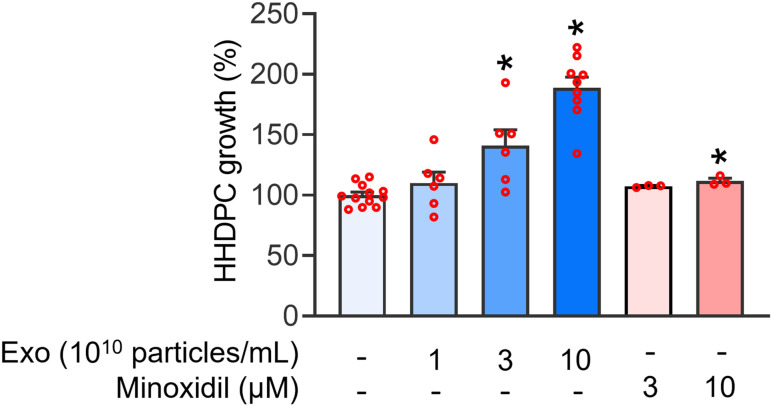
UC-MSC-Es enhance the proliferation of human hair dermal papilla cells. HHDPCs (2 × 10^3^ cells) were incubated with UC-MSC-Es (1 × 10^10^, 3 × 10^10^, and 1 × 10^11^ particles/mL) or minoxidil (3 and 10 μM) for 72 h. Cell proliferation was determined using the WST-1 assay. Data are expressed as the mean ± SEM (n = 3 - 12). *p < 0.05, compared with the control.

Hair dermal papilla cells play a significant role in hair growth. The impact of UC-MSC-Es on the proliferation of HHDPCs was evaluated. Fig 2 shows that UC-MSC-Es, at concentrations of 1 × 10^10^, 3 × 10^10^, and 1 × 10^11^ particles/mL, induced an increase in the proliferation of HHDPC in a concentration-dependent manner. Minoxidil (3 and 10 μM) was used as a positive control. The UC-MSC-Es demonstrate a significantly enhanced effect compared to minoxidil (Fig 2).

### UC-MSC-Es regulate cell cycle in HHDPCs

**Fig 3:**
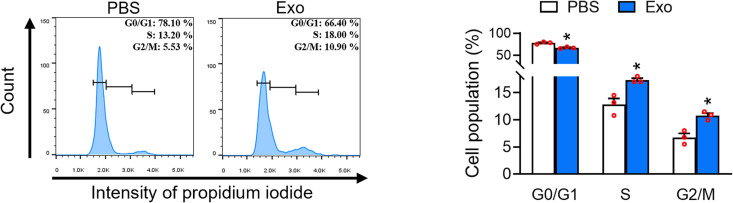
UC-MSC-Es promote the cell cycle in HHDPC. HHDPCs (2 x 10^4^) were incubated with PBS or UC-MSC-Es (1 × 10^11^ particles/mL) for 48 h. Cell cycle distribution was assessed using propidium iodide staining and measured by flow cytometry. Quantified flow cytometry results showed cell percentages in the G0/G1, S, and G2/M phases. Data are expressed as the mean ± SEM (n = 3). *p < 0.05, compared with the control.

To understand UC-MSC-Es’ effects on HHDPC proliferation, we examined the cell cycle distribution by treating HHDPCs with UC-MSC-Es at a concentration of 1 × 10^11^ particles/mL for 48 h. The treatment groups exhibited a notable decrease in the cell population in the G1 phase, accompanied by an increase in cell populations in the S and G2/M phases (Fig 3). These findings suggest that UC-MSC-Es-induced proliferation of HHDPCs was mediated by promoting S and G2/M phases.

### Modulation of cell cycle-associated protein expression by UC-MSC-Es

**Fig 4:**
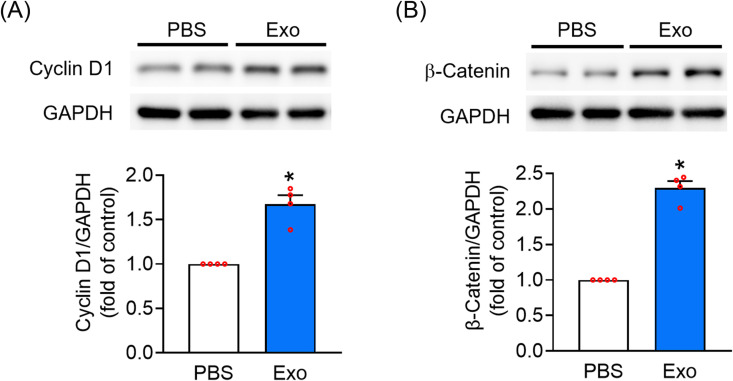
UC-MSC-Es elevate cyclin D1 and β-catenin expression in HHDPCs. HHDPCs (1 x 10^6^ cells) were incubated with PBS or UC-MSC-Es (1 × 10^11^ particles/mL) for 48 h. (A) Cyclin D1 and (B) β-catenin were analyzed by western blotting. The upper panel displays representative images, while the lower panel shows the quantified protein analysis results. Data are expressed as the mean ± SEM (n = 4). *p < 0.05, compared with the control.

The β-catenin signaling pathway plays a crucial role in the proliferation of HHDPCs [21–23]. Cyclin D1, along with cyclin-dependent kinases, acts as a mitogenic sensor and signaling transducer during the transition from G1 to S phase. This role of cyclin D1 is also influenced by the β-catenin signaling pathway [24]. Treatment with UC-MSC-Es (1 × 10^11^ particles/mL) for 48 h significantly increased the expression of both cyclin D1 and β-catenin in HHPDCs (Fig 4). These findings indicate the integral role of cyclin D1 and β-catenin in UC-MSC-Es-mediated cell proliferation.

### UC-MSC-Es increase Akt and GSK-3β in HHDPCs

**Fig 5:**
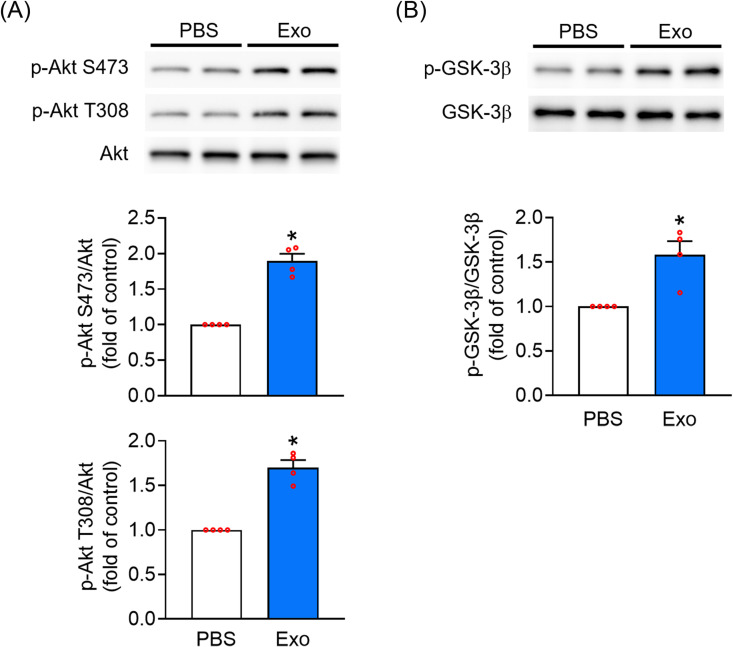
UC-MSC-Es elevate Akt and GSK3-β phosphorylation in HHDPCs. HHDPCs (1 x 10^6^ cells) were exposed to PBS or UC-MSC-Es (1 × 10^11^ particles/mL) for 24 h. The phosphorylation and total levels of (A) Akt and (B) GSK-3β were determined by immunoblotting, using antibodies that recognize phosphorylated and total forms of Akt and GSK-3β. The upper panel shows the representative images of immunoblots, while the lower panel shows the quantified results of protein bands. Data were shown as mean ± S.E.M. (n = 4). *p < 0.05, compared with the control.

The Akt and GSK-3β signaling pathways play a crucial role in the growth of HHDPCs [25–28]. Akt activation induces the phosphorylation and subsequent inactivation of GSK-3β at serine 9, stabilizing β-catenin and amplifying its transcriptional activity [29]. We tested whether Akt and GSK-3β are involved in modulating HHDPC proliferation in response to UC-MSC-Es treatment. Treatment with UC-MSC-Es (1 × 10^11^ particles/mL) for 24 h elevated Akt S473, Akt T308, and GSK-3β phosphorylation (Fig 5). These findings indicate that Akt and GSK-3β play a significant role in the UC-MSC-Es-mediated proliferation of HHDPCs.

### Role of PI3K and Akt signaling in UC-MSC-Es-induced cell proliferation of HHDPCs

**Fig 6:**
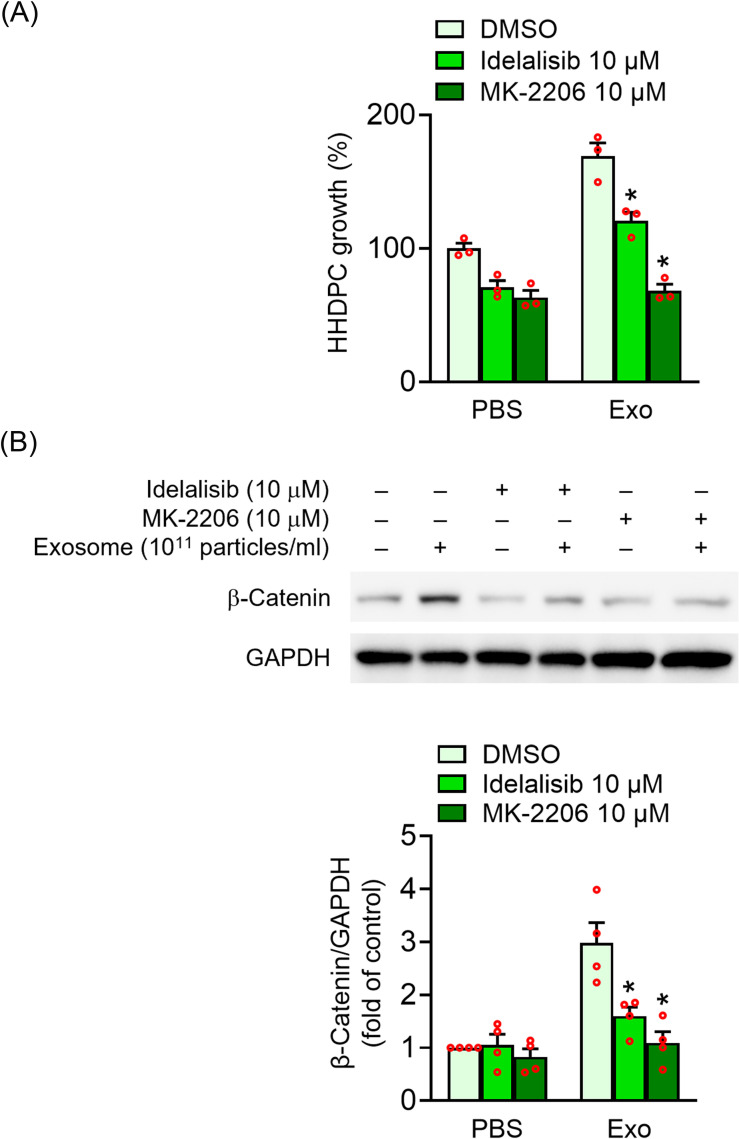
PI3K and Akt inhibitors attenuate UC-MSC-Es-induced HHDPC proliferation. HHDPCs (2 × 10^3^ cells) were pretreated with idelalisib (10 µM) or MK-2206 (10 µM) for 1 h and then treated with either PBS or UC-MSC-Es (1 × 10^11^ particles/mL) for 72 h. (A) Cell proliferation was assessed using the WST-1 assay. (B) The phosphorylation and total levels of β-catenin were determined by immunoblotting, using antibodies that recognize phosphorylated and total forms. Cell number was assessed via the WST-1 assay. Data were shown as mean ± S.E.M. (n = 3 - 4). *p < 0.05, compared with the control.

Idelalisib, a PI3K inhibitor, and MK-2206, an Akt inhibitor, were used to investigate the role of the PI3K and Akt pathways in UC-MSC-Es-mediated proliferation of HHDPCs. The treatment of idelalisib or MK-2206 significantly decreased UC-MSC-Es-induced cell growth (Fig 6A). Idelalisib and MK-2206 reduced HHDPC growth by 28.5% and 59.5% in the UC-MSC-Es treatment group compared with the control group. Furthermore, both idelalisib and MK-2206 significantly reduced the increase in β-catenin expression induced by UC-MSC-Es (Fig 6B). These findings suggest that the upregulation of β-catenin is mediated through the PI3K/Akt signaling pathway, supporting its role in the downstream effects of UC-MSC-Es on the proliferation of HHDPCs.

## Discussion

Hair loss can significantly impact a person’s psychological well-being [30]. A variety of treatment options for hair loss include topical treatments, such as anthralin, calcipotriol, tacrolimus, and corticosteroids, as well as systemic therapies like minoxidil, methotrexate, and cyclosporine. However, some treatments have notable side effects, including lymphadenopathy, folliculitis, and hypertrichosis [31]. There is a significant need for safer therapies to treat hair loss. HHDPCs play a crucial role in inducing the formation of new hair follicles. Recent research indicated that exosomes isolated from various sources, including adipose-derived stem cells [32], dermal papilla cells [33], human hair outer root sheath cells, and platelet-lysate exosomes [34], have the potential to stimulate HHDPC proliferation. UC-MSC-Es have demonstrated potential in tissue regeneration, particularly in improving neuronal survival and promoting functional recovery in optic nerve injuries [35]. Furthermore, these exosomes have been found to accelerate wound healing, especially when combined with hydrogels by reducing inflammation and promoting angiogenesis and tissue proliferation [36]. These findings emphasize the wide-ranging therapeutic potential of UC-MSC-Es in various medical applications. In the current study, UC-MSC-Es were found to enhance the proliferation of HHDPCs (Fig 2). Analysis of cell cycle distribution showed that UC-MSC-Es promote progression through the G1 phase, leading to an increased accumulation of cells in the S and G2/M phases, thus contributing to the enhanced proliferation of HHDPCs (Fig 3).

The PI3K, Akt, GSK-3β, and β-catenin signaling pathways significantly influence hair growth by regulating the activity and proliferation of hair follicle cells. PI3K and its direct downstream molecule, Akt, are crucial for promoting the proliferation of various cell types, including dermal papilla cells and keratinocytes [37,38]. Phosphorylation of Akt at serine 473 and threonine 308, which signifies Akt activation, triggers subsequent phosphorylation of the downstream protein GSK3β at serine 9. This cascade promotes β-catenin activity and upregulates cyclin D1, ultimately fostering enhanced hair follicle development and cell proliferation [24,39].

Much evidence reveals that the activation of Akt plays a central role in hair growth. The water extract of *Cacumen platycladi* promotes hair growth by stimulating the proliferation and migration of dermal papilla cells through the phosphorylation of Akt and GSK3β, leading to the accumulation of β-catenin and cyclin D1, which are essential for hair follicle development [40]. Similarly, ginsenoside Rg4 from ginseng increases the viability and size of dermal papilla spheres, activates PI3K/Akt signaling, and inhibits GSK3β activity, subsequently activating the β-catenin for hair growth [41]. Additionally, the Akt/GSK3β signaling pathway has been shown to restore and regulate hair follicle stem cell activity via β-catenin [42]. In this study, UC-MSC-Es treatment led to a marked increase in cyclin D1 and β-catenin expression in HHDPCs (Fig 4). Additionally, UC-MSC-Es treatment elevated the phosphorylation of Akt at S473 and T308 and GSK-3β at serine 9 (Fig 5).

Exosomes derived from various types of stem cells, including dermal papilla cells and hypoxia-preconditioned hair follicle mesenchymal stem cells, are increasingly recognized for their role in promoting hair growth through the activation of the PI3K/Akt signaling pathway. Notably, exosomes from hypoxia-preconditioned hair follicle mesenchymal stem cells enhance regenerative therapies by modulating the PI3K/Akt/mTOR pathway, which alleviates oxidative stress and inflammation, indirectly benefiting hair follicle health [43]. Furthermore, dermal papilla cells-derived exosomal miRNAs, including miR-181a-5p, activate Akt and Wnt/β-catenin pathways, encouraging stem cell proliferation and reducing apoptosis to facilitate hair growth [44]. These studies highlight the potential of exosome therapy to enhance hair restoration by modulating the PI3K/Akt pathway, which supports hair growth.

## Conclusions

**Fig 7:**
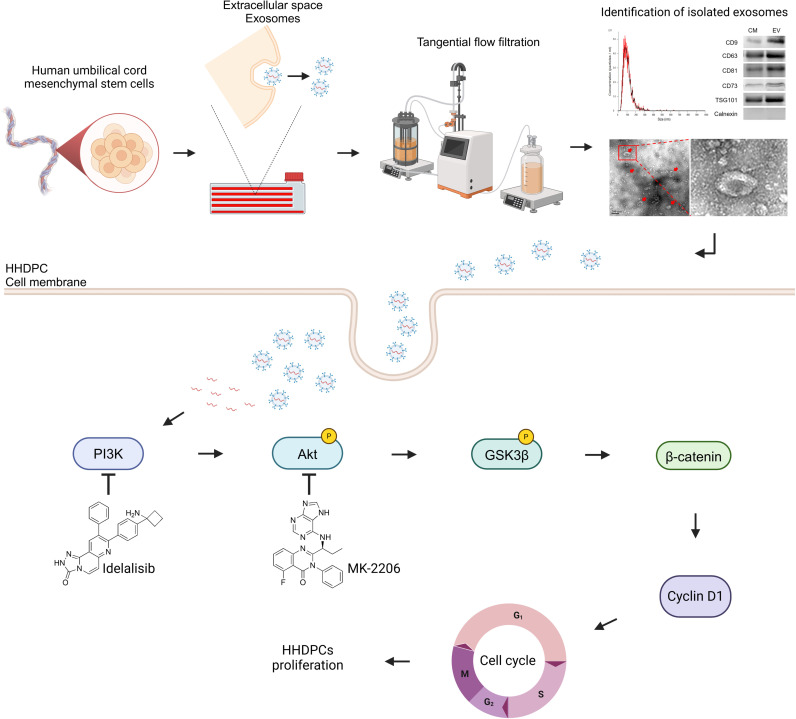
A schematic overview of UC-MSC-Es preparation and their effects on HHDPC proliferation. Human umbilical cord mesenchymal stem cell-derived exosomes (UC-MSC-Es) were concentrated from the culture medium and then applied to human hair dermal papilla cells (HHDPCs). The UC-MSC-Es facilitated the proliferation of HHDPCs by activating the PI3K and Akt signaling pathways. This activation resulted in the phosphorylation (inactivation) of GSK3β, which subsequently activated β-catenin and led to the upregulation of the cell cycle-related protein cyclin D1. The figure was created using BioRender (https://BioRender.com) under the appropriate license.

In conclusion, we highlight the considerable therapeutic potential of UC-MSC-Es in promoting hair growth. UC-MSC-Es significantly enhanced the proliferation of HHDPCs by activating the PI3K and Akt signaling pathways (Fig 7). These findings indicate that UC-MSC-Es may provide a new alternative to conventional hair loss treatments, presenting promising opportunities for future research and clinical applications in hair regeneration.

## Supporting information

S1 Raw Data Raw data for statistical analysis and figure generation. (XLSX)

S1 Raw Images Raw western blot scans. (PDF)
