# Supplementary figures and images for: Exosomes from human umbilical cord mesenchymal stem cells promote the growth of human hair dermal papilla cells
Source: PLoS One. 2025 Apr 30;20(4):e0320154. doi: 10.1371/journal.pone.0320154 (PMC12043141; doi:10.1371/journal.pone.0320154)

**Supplementary Figure 1 :**

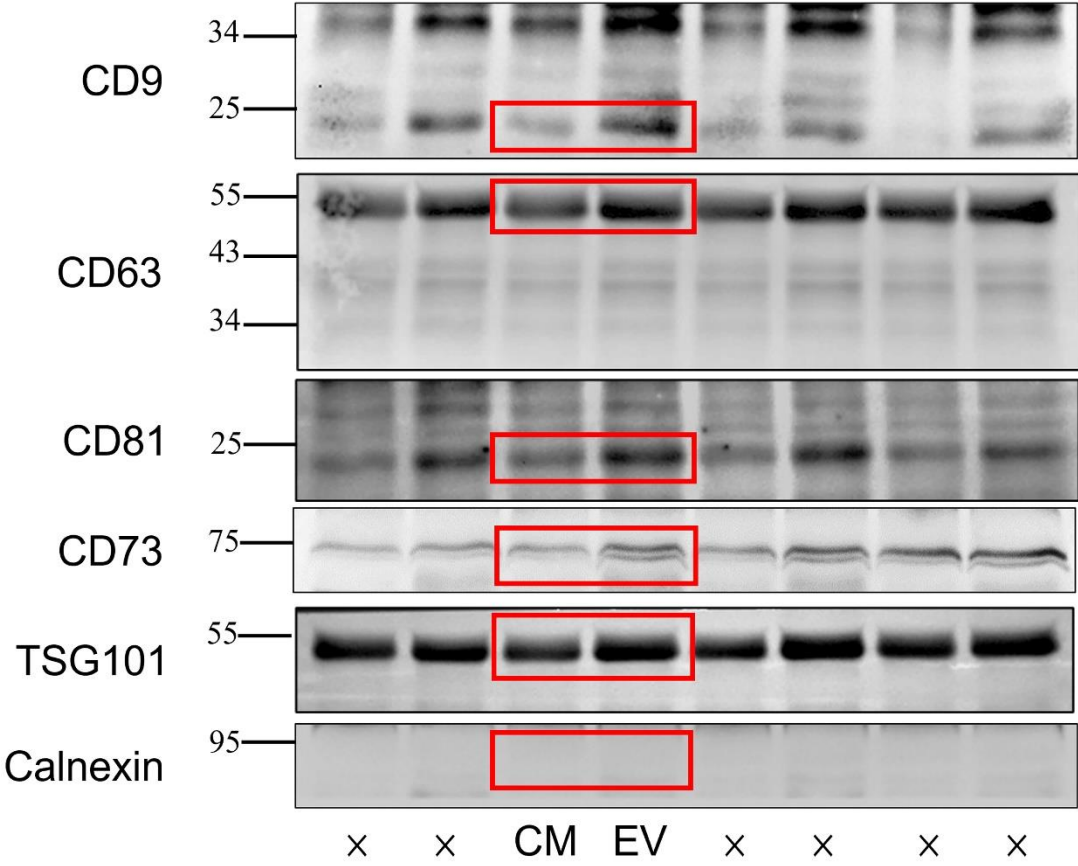

Supplementary Figure 4 :

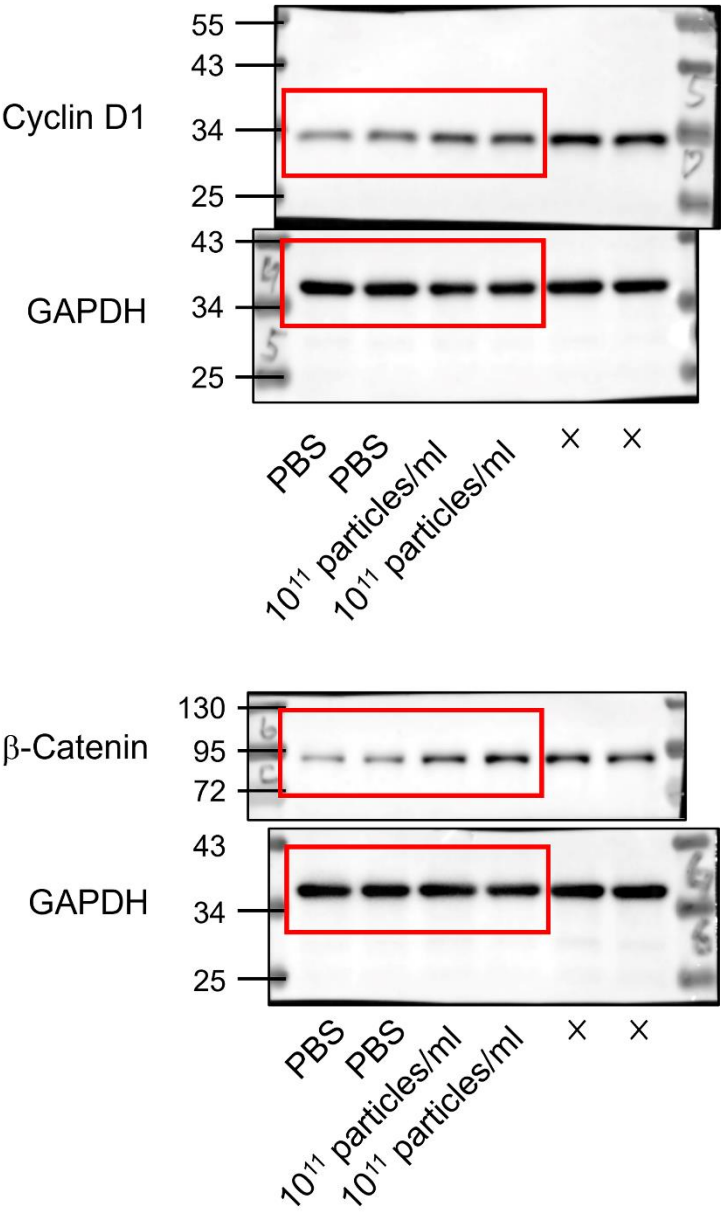

**Supplementary Figure 5 :**

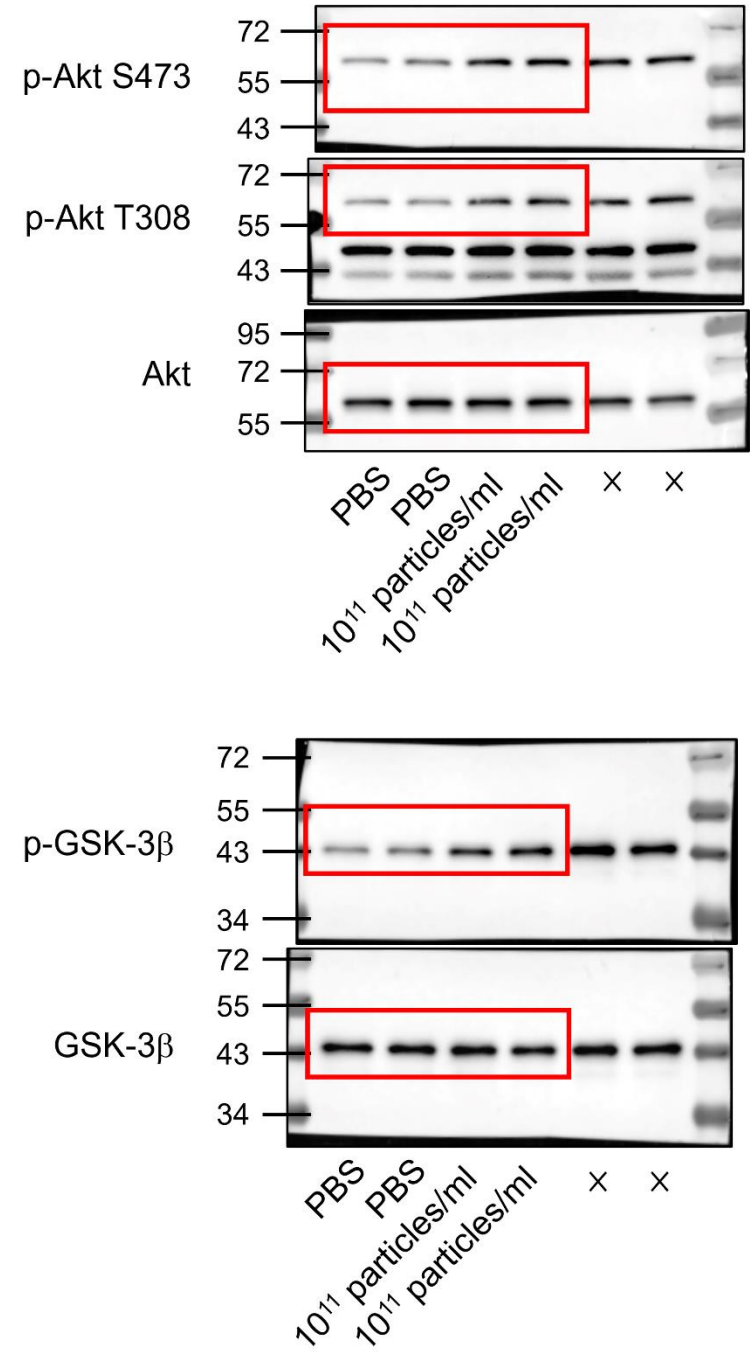

**Supplementary Figure 6 :**

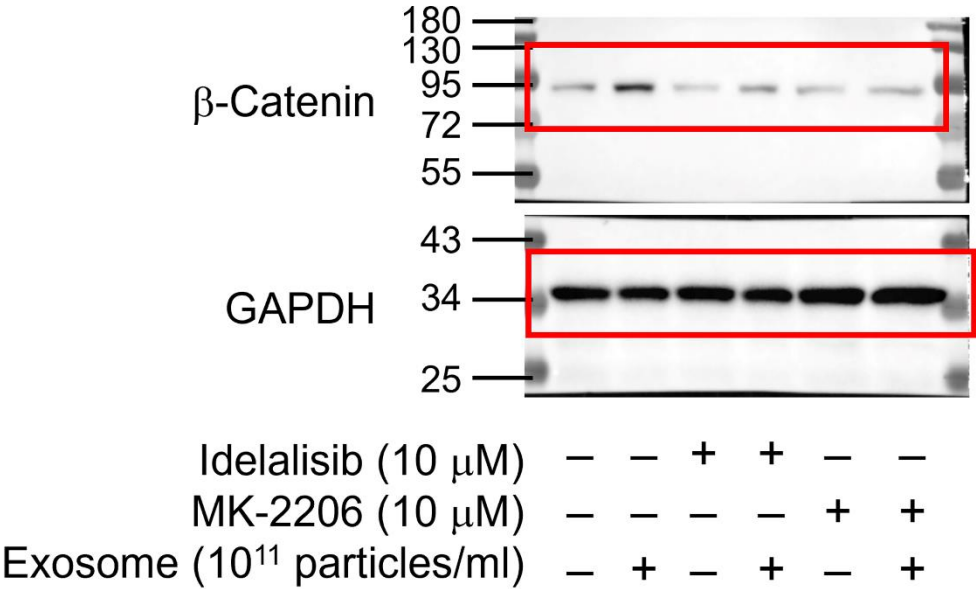

Supplement: S1 Raw Images — (PDF) [file pone.0320154.s002.pdf]
